# Cognitive functions in euthymic bipolar I and II patients: a cross sectional study

**DOI:** 10.3389/fpsyt.2025.1659408

**Published:** 2025-11-14

**Authors:** Rifat Serav Ilhan, Safiye Zeynep Tatli, Hilal Demirel, Vesile Şentürk Cankorur

**Affiliations:** 1 Department of Psychiatry, Ankara University Faculty of Medicine, Ankara, Türkiye; 2 Department of Psychiatry, Ankara Etlik City Hospital, Ankara, Türkiye; 3 Private Practitioner, Ankara, Türkiye

**Keywords:** bipolar I disorder, bipolar II disorder, cognitive impairment, verbal memory, euthymia

## Abstract

**Introduction:**

This study aimed to compare the neurocognitive profiles of euthymic patients with bipolar I (BD-I) and bipolar II (BD-II) disorder and healthy controls, while controlling for confounding clinical and pharmacological variables, to determine whether observed cognitive differences reflect true subtype distinctions or are secondary to illness burden.

**Methods:**

We assessed 78 clinically stable outpatients with BD-I or BD-II and 40 healthy controls using a comprehensive neuropsychological battery that included tests of verbal episodic memory, executive functioning, processing speed, attention, and working memory. All patients were in euthymia and receiving stable monotherapy. Analyses of covariance (ANCOVA) were conducted to compare group performance, adjusting for age, illness duration, number of depressive episodes, hospitalizations, and chlorpromazine-equivalent doses of antipsychotics and mood stabilizers.

**Results:**

Both BD subtypes demonstrated significant cognitive impairments relative to controls. BD-I patients showed broader and more severe deficits, especially in verbal episodic memory and executive flexibility. Importantly, only verbal memory impairments remained significant after full covariate adjustment, indicating a potential trait-like vulnerability in BD-I. Differences in executive function, processing speed, and attention between BD-I and BD-II were primarily explained by illness severity and medication exposure. Verbal episodic memory represents a robust and subtype-specific cognitive impairment in BD-I, whereas other cognitive differences between BD-I and BD-II are primarily attributable to modifiable clinical factors.

**Discussion:**

These findings underscore the importance of integrating cognitive evaluation into routine care and suggest that cognitive profiles may inform personalized interventions and diagnostic clarification in bipolar disorder.

## 1 Introduction

Bipolar disorder (BD) is a chronic psychiatric disorder marked by mood instability and recurrent depressive and (hypo)manic episodes, leading to functional impairment. It has two subtypes: bipolar I disorder (BD-I), with full manic episodes, and bipolar II disorder (BD-II), characterized by hypomanic episodes and major depression. The primary distinction between these subtypes is the severity of manic symptoms, but evidence suggests differences in course, treatment, and neurocognitive profiles.

Cognitive impairment in bipolar disorder is recognized as a core feature persisting beyond acute mood episodes, affecting psychosocial functioning, occupational performance, and quality of life (1, 2). Multiple meta-analyses have found moderate to severe impairments in executive function, verbal learning, memory, processing speed, and attention in euthymic patients, indicating trait-like cognitive abnormalities in BD (3, 4). The difference between BD-I and BD-II in cognitive performance remains unclear. Some studies suggest BD-I patients face broader and more severe cognitive deficits, especially in verbal episodic memory, executive functioning, and processing speed (5, 6). These deficits are linked to a more severe illness course in BD-I, including a higher prevalence of psychotic features, increased hospitalization rates, and more frequent manic episodes, which may contribute to cumulative neurobiological burden. Although earlier clinical models posited BD-II as a milder form of the illness, recent evidence suggests that many BD-II patients also demonstrate significant cognitive dysfunction, especially in executive domains and verbal memory (7, 8). However, these impairments may be less severe or more heterogeneous than those observed in BD-I (8, 9). These differing findings may stem from methodological differences, such as clinical status (euthymic vs. symptomatic), sample size, mood state at testing, and inadequate control for confounding variables, such as medication exposure, illness chronicity, and episode frequency. BD-I patients, often treated with antipsychotics and having experienced psychotic episodes or multiple hospitalizations, may have cognition affected by illness severity, potentially skewing group differences unless properly accounted for (10–12).

In line with previous literature, we hypothesized that:

1. Both BD-I and BD-II patients would show significant cognitive impairments compared to healthy controls.
2. BD-I patients would exhibit more pronounced deficits in verbal memory and executive function than BD-II, and these differences would persist after adjusting for clinical confounds.

We controlled for crucial clinical and pharmacological variables affecting cognitive function: chlorpromazine-equivalent antipsychotic dose, cumulative lithium and valproate load, number of hospitalizations, lifetime depressive episodes, and illness duration. By adjusting for these factors, we aimed to determine whether cognitive differences between subtypes persist after accounting for illness severity and treatment exposure.

## 2 Materials and methods

### 2.1 Participants

Adult outpatients (18–65 years) diagnosed with bipolar I (BD-I) or bipolar II (BD-II) disorder were recruited from Ankara University Psychiatry Clinic. Two board-certified psychiatrists confirmed diagnoses per DSM-V criteria. Patients needed to be in euthymia, defined as two months without significant mood symptoms (HDRS-17 score <10 and YMRS score <7). Additional inclusion criteria was stable pharmacotherapy (no medication changes for ≥6 weeks) and sufficient literacy (minimum level of education must be eight years) for testing, having an established diagnosis of BD-I or BD-II through clinical interview, participants must have enough hours of sleeping before testing.

Exclusion criteria were: neurological illness (e.g., stroke, epilepsy), intellectual disability (IQ<70), current substance use, other primary DSM V comorbid disorders (notably borderline personality disorders), recent electroconvulsive therapy or long-acting antipsychotic use (within past 6 months), taking of any kind of benzodiazepine and other CNS inhibituary medications effecting cognitive functions, severe uncontrolled medical disease (e.g., diabetes with complications, cardiovascular disease), and significant sensory or motor deficits, any transient physiological and endocrinological condition that have potential to effect cognitive test performances (etc. fasting, hypoglycemia, physical exercise, menstrual period, alcohol or caffeine intake). All participants had normal or corrected vision and could comply with testing procedures.

### 2.2 Instruments

Depressive and manic symptoms at screening were rated with the 17-item Hamilton Depression Rating Scale (HDRS-17) and Young Mania Rating Scale (YMRS), respectively. Both scales are widely used clinician-administered measures of depression and mania severity. Cognitive function was assessed using a comprehensive battery of neuropsychological tests, all of which were Turkish-validated versions. General intellectual ability, working memory, and processing speed were estimated from four subtests of the Wechsler Adult Intelligence Scale–Revised (WAIS-R): Information, Arithmetic, Digit Symbol, and Block Design. Verbal episodic memory was assessed by the Logical Memory subtest of the Wechsler Memory Scale–Revised (WMS-R) (13, 14). Executive function and cognitive flexibility were evaluated using the Wisconsin Card Sorting Test (WCST), which scores perseverative errors and the number of categories completed. All cognitive measures employed standard administration procedures and normative references, and their Turkish adaptations have demonstrated adequate reliability and validity (15).

### 2.3 Procedure

The neuropsychological battery was administered by a trained psychologist in a quiet testing room. Testing sessions took place during morning hours to minimize fatigue and diurnal variation, and each session lasted approximately 2–3 hours (including breaks as needed). Mood ratings (HDRS-17, YMRS) were again recorded at testing to document euthymia. All instructions and scoring followed the standardized manuals for each instrument.

### 2.4 Statistical analysis

Data were analyzed using IBM SPSS Statistics, version 22. Demographic and clinical variables were compared between BD-I and BD-II groups: categorical variables by chi-square tests and continuous variables by independent-samples t-tests (normal data) or Mann–Whitney U tests (non-normal data). Normality was assessed with the Shapiro–Wilk test, and variances with Levene’s test. Comparison of three groups’ cognitive outcomes were evaluated with analysis of variance (ANOVA) across neuropsychological measures. If the overall ANOVA was significant, follow-up univariate ANCOVAs were performed for each cognitive score. ANCOVA covariates included age, education, illness duration, psychiatric hospitalizations, major depressive episodes, chlorpromazine-equivalent antipsychotic daily dose, and cumulative mood stabilizers (lithium and valproate) – based on their known influence on cognition. Statistical significance was p<0.05.

### 2.5 Ethics

The study protocol was approved by the Ankara University Faculty of Medicine Clinical Research Ethics Committee. All participants provided written informed consent after receiving a complete description of study procedures. The research was conducted in accordance with the ethical standards of the Declaration of Helsinki.

## 3 Results

A total of 115 participants were included in the study: 56 with Bipolar I Disorder (BD-I), 24 with Bipolar II Disorder (BD-II), and 35 healthy controls. The BD-I, BD-II, and control groups were similar in basic demographic variables. The mean age was approximately 37 years in all three groups (BD-I: 37.7 ± 11.6; BD-II: 35.3 ± 11.1; controls: 37.8 ± 9.3 years), and the groups did not significantly differ in age (ANOVA *F* = 0.10, p = 0.63) or in years of education (BD-I: 12.4 ± 4.1; BD-II: 13.1 ± 2.7; controls: 12.9 ± 2.8, *F* = 0.55, p = 0.58). The sex distribution (female/male) was also comparable across groups (chi-square test, p > 0.5). All patients were in a euthymic state at the time of assessment, with mean HAM-D scores of 3.1 ± 3.7 for BD-I and 4.5 ± 2.6 for BD-II, and mean YMRS scores of 1.4 ± 2.6 for BD-I and 0.4 ± 1.1 for BD-II. There were no significant differences between the BD-I and BD-II groups on these mood scale scores (p = 0.21 for HAM-D; p = 0.20 for YMRS). As expected, both patient groups had slightly higher HAM-D and YMRS ratings than the healthy controls (who had mean HAM-D ~1.7 and YMRS ~0), but all scores were well below clinical thresholds, confirming the euthymic status.

Despite their similar current mood status, the BD-I and BD-II groups differed in certain characteristics of their illness history. BD-II patients had experienced a significantly higher number of depressive episodes on average than BD-I patients (mean ± SD: 4.3 ± 3.8 vs. 2.4 ± 2.9, respectively; *p* = 0.01). BD-I patients also had significantly more psychiatric hospitalizations than BD-II patients (1.1 ± 1.0 vs. 0.1 ± 0.3; *p* < 0.05). The two bipolar groups had a similar average duration of illness (approximately 10–11 years since diagnosis; *p* = 0.76) and a comparable mean age at onset of mood disorder (BD-I: 26.3 ± 9.5 years; BD-II: 24.5 ± 7.8 years, *p* = 0.41). Clinical and sociodemographic characteristics of BD-I and BD-II patients are summarized in **Table 1**.

**Table 1:** Sociodemographic and clinical characteristics of the BD-I, BD-II, and control groups.

Characteristic	Bipolar I (n = 56)	Bipolar II (n = 24)	Control (n = 35)	Test statistic (ANOVA/χ²/T test )	*p*-value
Age, years (Mean ± SD)	37.7 ± 11.6	35.3 ± 11.1	37.8 ± 9.3	F = 0.10	0.63
Education, years (Mean ± SD)	12.4 ± 4.1	13.1 ± 2.7	12.9 ± 2.8	F = 0.58	0.56
Age at illness onset (Mean ± SD)	26.3 ± 9.5	24.5 ± 7.8	–	F = 0.69	0.41
Illness duration, months (Mean ± SD)	134.6 ± 113.3	126.7 ± 93.5	–	F = 0.27	0.76
HAM-D score (Mean ± SD)	3.1 ± 3.7	4.5 ± 2.6	1.7 ± 1.6	–	0.21
YMRS score (Mean ± SD)	1.4 ± 2.6	0.4 ± 1.1	0 ± 0	–	0.20
No. of depressive episodes (Mean ± SD)	2.4 ± 2.9	4.3 ± 3.8	–	F = 5.80	0.01
No. of hospitalizations (Mean ± SD)	1.1 ± 1.0	0.1 ± 0.3	–	F = 24.1	< 0.001
Monotherapy treatment					
– Lithium, % (n)	32.1% (17)	16.7% (4)	–	–	–
– Valproic Acid, % (n)	37.7% (20)	4.2% (1)	–	–	–
– Lamotrigine, % (n)	0% (0)	37.5% (9)	–	–	–
– Atypical Antipsychotic, % (n)	30.2% (16)	41.7% (10)	–	–	–

All bipolar patients were receiving a single mood stabilizing medication. The distribution of monotherapy treatments is summarized in **Table 1**. In the BD-I group, 32.1% (n = 17) were on lithium monotherapy, 37.7% (n = 20) on valproic acid, and 30.2% (n = 16) on an atypical antipsychotic. In the BD-II group, 16.7% (n = 4) were on lithium, only 4.2% (n = 1) on valproic acid, 37.5% (n = 9) on lamotrigine, and 41.7% (n = 10) on an atypical antipsychotic. Thus, BD-I patients were more often treated with classical mood stabilizers (lithium/valproate), whereas BD-II patients more frequently received lamotrigine or a single atypical antipsychotic. The average doses and blood levels (where applicable) of these medications were within therapeutic ranges (for example, mean lithium level ~0.66 mEq/L in those on lithium). There were no indications of active side effects such as oversedation at the time of cognitive testing.

### 3.1 Cognitive performance in BD-I and BD-II

A series of one-way analyses of variance (ANOVAs) was conducted to compare the three groups – Bipolar I (BD-I), Bipolar II (BD-II), and healthy controls – on each cognitive measure. *Post hoc* pairwise comparisons were conducted using the Fisher LSD test or the Games–Howell test (when variance homogeneity assumptions were violated) to determine which group differences were significant. Descriptive statistics and ANOVA outcomes for each cognitive domain are presented in **Table 2**. Detailed pairwise comparison results are provided in **Table 3**. A final ANCOVA model was also tested for verbal memory, incorporating key clinical covariates, and its summary is given in **Table 4**.

**Table 2:** Descriptive statistics and one-way ANOVA results by cognitive domain.

Cognitive measure	BD-I (Mean ± SD)	BD-II (Mean ± SD)	Control (Mean ± SD)	ANOVA F _(df=2, _)	p	Partial η²	Post hoc group differences
Premorbid IQ (General Knowledge)	11.2 ± 3.1	13.1 ± 5.0	11.7 ± 3.4	2.5	.080	.04	BD-I = BD-II = Control
Executive function							
WCST Categories Completed	4.9 ± 1.7	5.2 ± 1.4	5.9 ± 0.1	5.9	.004*	.09	BD-I = BD-II < Control
WCST Perseverative Errors	19.4 ± 17.5	13.9 ± 14.5	7.3 ± 2.8	8.0	.001*	.12	BD-I < Control; BD-II = Control (BD-I = BD-II)
WCST Non-perseverative Errors	11.4 ± 11.5	14.4 ± 8.9	7.3 ± 2.8	5.7	.005*	.09	BD-I = BD-II < Control
Verbal memory							
WMS Logical Memory (Immediate)	10.4 ± 3.6	14.4 ± 4.7	17.2 ± 4.9	26.5	<.001*	.30	BD-I < BD-II < Control
WAIS core subtests							
Block Design	29.2 ± 8.3	32.1 ± 7.5	29.6 ± 6.9	7.05	.001*	.10	BD-I = Control < BD-II
Digit Symbol Coding	65.8 ± 12.0	71.8 ± 9.5	69.5 ± 10.8	3.98	.022*	.06	BD-I < BD-II; BD-I < Control; BD-II = Control
Arithmetic	10.5 ± 3.2	10.8 ± 2.9	11.0 ± 3.1	0.54	.585	.01	BD-I = BD-II = Control

BD-I , Bipolar I disorder; BD-II , Bipolar II disorder; WCST , Wisconsin Card Sorting Test; WMS , Wechsler Memory Scale (Logical Memory Immediate Recall); WAIS , Wechsler Adult Intelligence Scale. Higher scores indicate better performance for all measures except WCST Errors, where higher scores indicate worse performance (more errors). Partial η² , effect size for the group factor from ANOVA. *Post hoc* group differences are summarized using Fisher’s LSD test (except Block Design and Coding, which used Games–Howell). “<” indicates significantly worse performance (lower scores or, for error counts, higher errors) compared to the group on the right side of the symbol. For example, “BD-I , BD-II < Control” means BD-I and BD-II did not differ from each other, and both performed worse than (significantly below) the Control group. For perseverative errors, BD-II did not differ significantly from controls, whereas BD-I made significantly more errors than controls (and BD-I vs BD-II was non-significant).

**Table 3:** Pairwise *Post Hoc* comparisons for cognitive measures (mean differences and significance).

Measure	BD-I vs BD-II	BD-I vs control	BD-II vs control
Premorbid IQ (General Knowledge)	–1.9 (p = .17)	–0.5 (p = .67)	+1.4 (p = .09)
WCST Categories Completed	–0.7 (p = .36)	–1.0 (p = .012)	–0.7 (p = .045)
WCST Perseverative Errors	+5.5 (p = .15)	+12.1 (p = .010)	+6.6 (p = .12)
WCST Non-perseverative Errors	–3.0 (p = .22)	+4.1 (p = .037)	+7.1 (p = .003)
WMS Logical Memory (Immediate)	–4.0 (p <.001)	–6.8 (p <.001)	–2.8 (p = .001)
WAIS Block Design	–2.9 (p <.001)	–0.4 (p = .757)	+2.5 (p = .004)
WAIS Digit Symbol Coding	–3.9 (p = .006)	–1.6 (p = .029)	+2.3 (p = .609)
WAIS Arithmetic	–0.3 (p = .78)	–0.5 (p = .60)	–0.2 (p = .83)

Entries are mean differences (Group1 – Group2) from Fisher’s LSD *post hoc* tests, or Games–Howell where applicable (Block Design, Coding). Values in parentheses are the two-tailed significance levels for the mean difference. A positive difference indicates that the first group listed (left) scored higher on the measure than the second group (right), whereas a negative difference indicates the first group scored lower. For example, for Logical Memory, “–4.0 (p <.001)” under BD-I vs BD-II indicates BD-I scored 4 points lower than BD-II on average, a difference significant at p <.001. Bold font or asterisks are not shown in the table, as p-values indicate significance: p <.05 is considered significant. BD-I, Bipolar I; BD-II, Bipolar II; WCST, Wisconsin Card Sorting Test; WMS, Wechsler Memory Scale; WAIS, Wechsler Adult Intelligence Scale.

**Table 4:** Final ANCOVA model for verbal memory (WMS Logical Memory Immediate Recall), controlling for illness severity and medication covariates.

Covariates	df (*n*)	F	p	Partial η²
Group (BD-I, BD-II, Control)	2, 60	13.28	<.001**	.149
Number of hospitalizations (lifetime)	1, 60	0.84	.364	.014
Number of depressive episodes	1, 60	0.51	.478	.008
Illness duration (years)	1, 60	1.20	.277	.020
Lithium exposure (current)	1, 60	0.17	.684	.003
Valproic acid exposure (current)	1, 60	0.30	.586	.005
Chlorpromazine equivalent dose (mg)	1, 60	2.55	.114	.032
*Model R² = .19 (adjusted R² = .11)*				

The table shows the results of a one-way ANCOVA comparing diagnostic groups (BD-I, BD-II, Control) on verbal memory, while controlling for key covariates related to illness severity and treatment. Partial η² represents the effect size (proportion of variance explained) for each predictor. Group remains a significant predictor of memory performance after controlling for all covariates, with BD-II patients scoring higher than BD-I patients by an estimated 4.3 points (adjusted mean difference, *p* = .004). None of the covariates had a significant influence on memory (all p >.10), indicating that differences in hospitalizations, episodes, illness chronicity, or medication exposure do not fully account for the cognitive differences between groups. The model explains approximately 19% of the variance in verbal memory scores (adjusted R² ~ 11%). BD-I, Bipolar I; BD-II, Bipolar II; num, numerator; den, denominator; R², proportion of variance explained by the model.

### 3.2 Pattern of cognitive performance across groups

#### 3.2.1 Premorbid intellectual ability

Premorbid IQ was evaluated by WAIS-R general information sub-scale. The groups did not differ on the general information index (all *p* > 0.05).

#### 3.2.2 Executive functioning (Wisconsin Card Sorting Test)

*Completed categories:* Both BD-I and BD-II completed significantly fewer categories than controls (BD-I, p = .012; BD-II: *p* = .045), but bipolar groups did not differ from each other (p=0.36). When chlorpromazine-equivalent and lithium doses, valproate doses, lifetime hospitalizations, and past depressive episodes were simultaneously controlled, the diagnostic-group effect on WCST categories was not significant (F < 0.01, p = 0.99), only antipsychotic load remained significant (*F* = 7.07, *p* = .010, partial η² = .087).

Perseverative errors. In Unadjusted ANOVA analysis showed that BD-I group had more perseverative errors than controls (+12.1, *p* = .010); BD-II did not differ. After adjusting for chlorpromazine-equivalent, lithium, valproate doses, hospitalizations, and depressive-episode count in ANCOVA analysis, the diagnostic-group effect on WCST perseverative errors was insignificant (F = 0.20, p = .659, partial η² = .003). Only antipsychotic load remained significant (F = 4.06, p = .048, partial η² = .053),

Non-perseverative errors were elevated in both bipolar groups versus controls (BD-I: +4.1, *p* = .037; BD-II: +7.1, *p* = .003) but bipolar groups were not differed from each other. When chlorpromazine-equivalent, lithium, and valproate doses, together with lifetime hospitalizations and past depressive episodes, were entered into the ANCOVA simultaneously, the diagnostic-group effect on WCST non-perseverative errors was rendered non-significant (F = 1.22, p = .274).

#### 3.2.3 Verbal episodic memory (WMS Logical Memory)

A graded pattern emerged in ANOVA analysis: BD-I scored significantly lower than BD-II (p < 0.001) and controls ( p < 0.001); BD-II also performed poorly than controls ( p = .001). Verbal-memory impairment exists across subtypes but is most severe in BD-I, correlating with a higher illness burden. In a fully adjusted ANCOVA controlling for various factors, the diagnostic-group effect on the WMS logical memory performance remained significant (F = 10.62, p = .002, partial η² = .129) (**Table 4**).

#### 3.2.4 Processing speed and working memory

##### 3.2.4.1 WAIS arithmetic (working memory)

No significant differences were found across groups (*F* = 0.54, *p* = .585), and ANCOVA models confirmed this null finding. Working memory, as assessed via mental arithmetic, appears preserved across bipolar subtypes and controls.

##### 3.2.4.2 WAIS DIGIT symbol coding (processing speed)

BD-I performed worse than BD-II (p = .006) and controls (p = .029), with no significant difference between BD-II and controls. After adjustment, the diagnostic-group effect was non-significant (*F* = 0.78, *p* = .381, partial η² = .011); medication and clinical covariates were also non-significant.

#### 3.2.5 Visuospatial construction (WAIS Block Design)

WAIS Block Design (Visuospatial Construction): Unadjusted analyses showed a group effect, with BD-II outperforming BD-I and controls. In the adjusted model, the group effect was non-significant (*F* (2,60) = 2.81, *p* = .117).

The result of ANOVA and subsequent *post-hoc* analyses comparing BD-I, BD-II and control group are presented in **Tables 2** and **3**.

## 4 Discussion

### 4.1 Summary of key findings

This study analyzed neurocognitive profiles of patients with Bipolar I disorder (BD-I), Bipolar II disorder (BD-II), and healthy controls using various neuropsychological assessments. In the unadjusted analysis the study found bipolar groups showed significant cognitive impairments in the several cognitive domains. Specifically, BD-I patients had significant deficits in verbal episodic memory compared to BD-II and controls. In an immediate verbal recall task, BD-I patients performed lowest, BD-II patients were intermediate, and controls performed highest, indicating a gradient of impairment (BD-I < BD-II < Control). Executive functioning, measured by the Wisconsin Card Sorting Test (WCST), also showed group differences. Both bipolar groups demonstrated impaired concept formation and greater error rates compared to healthy controls. BD-I and BD-II patients completed fewer WCST categories on average than controls, indicating reduced overall executive problem-solving success. The two bipolar groups did not significantly differ from each other in number of categories completed. In terms of errors, both BD-I and BD-II groups made a higher total number of non-perseverative (random) errors than controls. Notably, perseverative errors – a specific indicator of set-shifting difficulty – were significantly elevated only in the BD-I group. BD-I patients committed more perseverative errors than healthy controls and also more than BD-II patients, whereas the BD-II group’s perseverative error count was similar to that of controls. This suggests that set-shifting deficits were present in BD-I but not evident in BD-II in the unadjusted analysis. Group differences were further observed in processing speed. On the WAIS Digit Symbol Coding test (psychomotor processing speed), there was a significant main effect of group. BD-I patients had the slowest processing speed, with a mean Coding score significantly lower than both the BD-II and control groups. In contrast, BD-II patients performed comparably to healthy controls on the Coding task, indicating that processing speed was largely intact in BD-II. A similar pattern emerged in the visuospatial domain: BD-I patients scored lowest on the Block Design test, significantly below the high performance of BD-II patients and controls. Short-term working memory performance, measured by the WAIS Arithmetic subtest, showed no significant differences across groups, indicating preserved function regardless of diagnosis. Additionally, premorbid intellectual ability, assessed through general knowledge, was similar among all groups.

In summary, prior to adjustments, BD-I was associated with broad cognitive deficits (especially in memory, executive function, and processing speed), whereas BD-II showed milder impairments and even relative strengths (normal visuo-spatial ability and processing speed).

To determine whether the observed group differences were independent of illness burden and medication effects, we conducted ANCOVA models for each cognitive outcome, controlling for chlorpromazine-equivalent antipsychotic dose, lithium load, valproate load, total number of lifetime psychiatric hospitalizations, and number of past depressive episodes. After adjusting for these covariates, most of the initial group differences were no longer statistically significant, suggesting that many cognitive disparities were attributable to differences in treatment or illness severity rather than inherent to diagnostic groups. Crucially, the only cognitive measure that remained significantly different between BD-I, BD-II, and the control group after full adjustment was verbal memory performance. In the fully adjusted ANCOVA model for the WMS performance (controlling for antipsychotic, lithium, valproate, hospitalizations, and depressive episodes), a robust effect of diagnostic group persisted. After adjustment, many cognitive differences diminished and became insignificant. BD-I, BD-II patients and controls exhibited no notable differences in executive function (e.g., WCST), processing speed, working memory, or visuospatial skills after controlling for medication exposure and clinical factors.

Across cognitive domains, current antipsychotic exposure was the most consistent covariate influencing performance, particularly on executive function tasks assessed by WCST subtests, such as perseverative errors and completed categories. Notably, verbal memory performance remained significantly associated with diagnostic group status after full statistical adjustment, suggesting that the observed cognitive impairments in bipolar groups was not entirely attributable to pharmacological or illness-related confounders.

### 4.2 Comparison with previous literature

Our findings align with existing research on cognitive impairments in bipolar disorder, particularly regarding differences between BD-I and BD-II. Prior studies indicate that both subtypes exhibit significant neurocognitive deficits compared to healthy individuals (6). Cognitive deficits span multiple domains—including verbal episodic memory, visual memory, attention, executive function, and processing speed—and persist during remission in both subtypes (16, 17). However, inconsistencies persist regarding the extent and nature of these deficits. Our comprehensive neuropsychological testing and covariate adjustments may help clarify these discrepancies.

### 4.3 Verbal episodic memory

We observed pronounced verbal episodic memory impairments, with BD-I patients exhibiting the most severe deficits, followed by BD-II, and healthy controls performing best (BD-I < BD-II < HC). This gradient aligns with literature identifying verbal memory as a core deficit in bipolar disorder, especially in BD-I (1, 5, 6, 10, 18–20). The meta-analytic evidence shows large effect sizes for verbal memory measures in BD-I and smaller but significant impairments in BD-II (6). The study results were in line with these findings, as bipolar groups showed significantly poorer performance on the WMS Logical Memory task, even after adjusting for clinical and treatment variables. Longitudinal evidence suggests that memory deficits in BD-I may worsen over time, potentially due to neurotoxic effects of manic episodes or psychosis (21), pointing to a neuropathological basis involving hippocampal or fronto-hippocampal dysfunction. This interpretation is supported by neurobiological evidence linking verbal memory deficits to structural and functional alterations in hippocampal-prefrontal circuits that appear to be present across bipolar subtypes, though potentially more pronounced in BD-I (11, 20).

### 4.4 Executive functions

Bipolar I and Bipolar II differ in the pattern and magnitude of executive dysfunction, but findings vary across samples and measures. Meta-analytic and comparative studies have indicated greater and more widespread executive deficits in BD-I, whereas BD-II shows smaller impairments (6, 11, 22). The meta-analyses have reported that euthymic bipolar patients had cognitive impairment in executive domains (23, 24). The present study's findings were in line with previous studies on executive function in BD-I and BD-II patients, as demonstrated by impaired Wisconsin Card Sorting Test (WCST) performance, with fewer categories completed and more non-perseverative errors compared to healthy controls. Only BD-I patients exhibited significant perseverative errors, suggesting greater cognitive inflexibility, whereas BD-II patients performed similarly to controls in this domain. However, after adjusting for clinical covariates—such as illness duration, hospitalizations, and antipsychotic exposure—executive function differences between BD-I and BD-II disappeared, indicating that these disparities reflect illness severity and treatment effects rather than intrinsic subtype differences (10–12, 24, 25).

The broader literature has provided mixed findings on the processing speed performance of bipolar patients. While some studies reported processing speed deficits in BD-II (26), others have found relatively preserved performance, particularly in euthymic patients with shorter illness duration [27]. The meta-analytic evidence suggested that processing speed impairments may be more consistently present in BD-I, with effect sizes varying considerably across studies for BD-II [6]. In the present study, processing speed, assessed via the Digit Symbol Coding task, was impaired in both BD-I and BD-II compared to controls, with BD-I initially slower than BD-II. This difference diminished after adjusting for covariates, notably antipsychotic dose and clinical factors. For working memory, both BD-I and BD-II performed within normative ranges on the WAIS Arithmetic subtest, and differences on the Digit Span test were non-significant post-adjustment, contrasting with prior reports of working memory impairment in bipolar patients (27, 28).

One of the most striking aspects of our findings was the dramatic reduction in cognitive differences between bipolar subtypes and controls after controlling for medication exposure and clinical variables. This observation has profound implications for interpreting the existing literature and aligns with emerging evidence suggesting that many apparent cognitive differences between BD-I and BD-II may be confounded by differential treatment patterns and illness characteristics.

The systematic review evidence supports this interpretation, with several studies reporting that controlling for antipsychotic exposure, lithium use, and illness severity substantially attenuates or eliminates cognitive differences between bipolar subtypes (12, 29). Our finding that antipsychotic exposure was the most consistent predictor of cognitive performance across domains is particularly important, as BD-I patients in our sample were more frequently treated with antipsychotic medications compared to BD-II patients, who more commonly received lamotrigine or lower-dose antipsychotic treatments.

This pattern reflects real-world clinical practice, where BD-I patients typically require more intensive pharmacological intervention due to the severity of manic episodes and higher rates of psychotic features. However, it raises important questions about whether cognitive differences attributed to diagnostic subtypes may actually reflect differential treatment effects. The meta-analytic evidence showing that lithium use was associated with larger effect sizes for cognitive flexibility and episodic memory further supports the critical importance of considering medication effects in cognitive research (6).

### 4.5 Clinical implications

Our findings, integrated with the broader literature, have several important clinical implications. First, the identification of verbal memory as a core deficit that persists despite controlling for medication and clinical factors suggests that memory assessment should be a priority in both BD-I and BD-II patients. The meta-analytic evidence supports the use of specific instruments, including the Trail Making Test, Hayling Test, Digit Span Total, and Category Fluency, as sensitive measures for cognitive assessment in bipolar disorder (6) Second, the substantial impact of medication on cognitive performance, particularly antipsychotic exposure, emphasizes the need for careful consideration of treatment effects when interpreting cognitive test results. Clinicians should be aware that cognitive deficits may partially reflect medication side effects rather than intrinsic illness features, particularly in BD-I patients who more commonly receive antipsychotic treatment. Third, the evidence for more selective cognitive impairments in BD-II suggests that cognitive rehabilitation interventions may need to be tailored to specific deficit profiles. While BD-I patients may benefit from comprehensive cognitive remediation targeting multiple domains, BD-II patients might benefit from more focused interventions targeting specific areas of impairment while leveraging preserved cognitive strengths.

### 4.6 Methodological considerations and limitations

The substantial heterogeneity observed in meta-analytic studies of bipolar cognition highlights the importance of methodological factors in interpreting cognitive differences between subtypes. Our study's comprehensive approach to controlling potential confounders may explain why we observed fewer significant differences between BD-I and BD-II compared to some previous studies. Several methodological factors likely contribute to inconsistent findings in the literature. the smaller representation of BD-II patients in most studies (our BD-II n=24 vs. BD-I n=56 reflects this common imbalance) may limit power to detect subtle differences or lead to unstable effect size estimates. Differences in diagnostic criteria, mood state at testing, and medication status across studies create substantial heterogeneity, complicating the interpretation of pooled analyses. The systematic review evidence indicates that studies testing patients during euthymic states tend to show smaller effect sizes and less pronounced differences between subtypes (8, 30). This is consistent with our findings, as all patients were rigorously assessed to ensure euthymic status at the time of testing. Additionally, the duration of euthymia may be critical, as some cognitive deficits may improve with sustained mood stability (31).

This study's strength is its focus on euthymic patients under monotherapy, reducing confounding factors from mood state and polypharmacy. We utilized validated cognitive tests and maintained strict statistical control for clinical variables—antipsychotic dose, lithium/valproate load, hospitalizations, and depressive episodes. Only verbal memory impairment in BD-I remained significant after adjustment, indicating a possible trait-like deficit.

This is the first study to systematically include chlorpromazine-equivalent antipsychotic dosage, lithium and valproate load, prior hospitalizations, and lifetime depressive episodes as covariates in analyzing neurocognitive performance in BD-I and BD-II populations. By uniformly applying this adjustment across domains like verbal memory, executive function, processing speed, and visuospatial construction, we distinguished robust cognitive deficits from those likely influenced by treatment exposure or illness burden.

Our study’s limitations comprise its cross-sectional design, medication-related confounding, moderate sample size (particularly for BD-II), and a cognitive test battery that could not cover all aspects of cognition. These limitations temper our conclusions but highlight areas for careful interpretation and inquiry. Future longitudinal studies with larger, stratified samples and broader assessments (e.g., neuroimaging, social cognition) are necessary to establish the stability and causality of cognitive deficits in bipolar subtypes (32, 33).

### 4.7 Future research directions

To clarify the trajectory and causes of cognitive decline in bipolar disorder, future studies should adopt longitudinal designs with repeated cognitive assessments, detailed medication tracking, and neurobiological measures (e.g., imaging, inflammation). This approach will determine whether deficits like verbal memory impairment in BD-I are stable traits or consequences of illness progression and treatment, while identifying critical intervention periods.

### 4.8 Conclusions

This study highlights cognitive impairment as a core feature of bipolar disorder, demanding clinical attention alongside mood symptoms. While BD-I shows more severe deficits—especially in verbal episodic memory—many differences between BD-I and BD-II diminish after accounting for illness severity and treatment. Verbal memory impairment in BD-I suggests a trait-level vulnerability. Clinically, personalized care is essential: BD-I may require targeted cognitive interventions, while BD-II patients benefit from depression management to prevent cognitive decline. Treating cognition as a vital sign can enhance recovery and quality of life. Future research should explore biological mechanisms and interventions to improve cognitive outcomes.

## Data Availability

The raw data supporting the conclusions of this article will be made available by the authors, without undue reservation.
